# Case report: A novel somatic *SDHB* variant in a patient with bladder paraganglioma

**DOI:** 10.3389/fendo.2024.1386285

**Published:** 2024-06-07

**Authors:** Thao Nguyen, Zehra Ordulu, Sunaina Shrestha, Urja Patel, Paul L. Crispen, Lisa Brown, Sara M. Falzarano, Hans K. Ghayee, Juan Pablo Perdomo Rodriguez

**Affiliations:** ^1^ Department of Medicine, University of Florida, Gainesville, FL, United States; ^2^ Department of Pathology, Immunology and Laboratory Medicine, University of Florida College of Medicine, Gainesville, FL, United States; ^3^ Division of Endocrinology, University of Florida, Gainesville, FL, United States; ^4^ Department of Urology, University of Florida, Gainesville, FL, United States; ^5^ Department of Genetics, University of Florida, Gainesville, FL, United States; ^6^ Health Cancer Center, University of Florida, Gainesville, FL, United States; ^7^ Division of Endocrinology, Malcom Randall Veterans Affairs (VA) Medical Center, Gainesville, FL, United States

**Keywords:** paraganglioma, bladder paraganglioma, *SDHB*, c.642G>A, p.Q214Q, leiomyoma

## Abstract

**Background:**

Paragangliomas (PGL) are rare neuroendocrine tumors derived from the autonomic nervous system paraganglia. Urinary bladder paragangliomas (UBPGL) originate from the sympathetic neurons of the urinary bladder wall and represent 0.7% of all paragangliomas and <0.05% of all bladder tumors. PGL and UBPGL can be associated with *SDHB, SDHD, NF1, and VHL* gene variants, with the most common germline alterations found in *SDHB* and *VHL*.

**Case report:**

We report a case of a 42-year-old woman who presented with menorrhagia/hematuria, uterine leiomyomas, as well as cardiac and bladder masses. The cardiac mass was favored to be a myxoma based on clinical findings, while the bladder mass was diagnosed as UBPGL. A novel *SDHB* mutation (c.642G>A, p Q214Q), detected in the UBPGL, was proven to be somatic. Although this variant was seemingly synonymous, it was predicted to have a loss of function due to the splice site effect, which was further supported by the immunohistochemical loss of *SDHB*.

**Conclusion:**

This case highlights the challenges of diagnosing an extremely rare entity, bladder paraganglioma, with an emphasis on the multidisciplinary approach to navigate various clinical and imaging findings that may initially be misleading. In addition, a novel loss of function *SDHB* variant that could have been overlooked as a synonymous variant is herein reported, while also illustrating the importance of both germline and somatic mutation testing.

## Introduction

Paragangliomas (PGLs) are rare neuroendocrine tumors derived from extra-adrenal chromaffin cells (1). The incidence of paragangliomas is often described together with the incidence of pheochromocytomas (PCCs), which is approximately 0.6 cases per 100,000 person-years (2). PGLs located in the neck and skull are usually parasympathetic and nonfunctional while those located in the thorax, abdomen, and pelvis tend to be sympathetic and hypersecretory (1). Urinary bladder paragangliomas (UBPGL) are extremely rare tumors originating from the sympathetic neurons of the urinary bladder wall, which represent 0.7% of all PGLs and <0.05% of all bladder tumors (3). UBPGLs may manifest with catecholamine excess, ranging from 55% to 91% of the cases (4–6) Diagnosing non-functioning UBPGLs can be challenging, as they may only manifest as hematuria or be detected incidentally from imaging studies. Consequently, up to two-thirds of UBPGLs are diagnosed following surgery or a biopsy (7).

Risk factors for metastatic UBPGLs include high levels of catecholamine excess, young age, and large tumor size (7).PGLs and UBPGLs can be associated with mutations involving *SDHB, SDHD, VHL*, and *NF1* (2). The former two genes, particularly *SDHB* are the most commonly mutated genes in patients with germline mutations (7). Previous research has illustrated that pathogenic *SDHB* variants in PGLs increase the risk of metastatic disease (7, 8).

## Case presentation

A 42-year-old female with menometrorrhagia secondary to presumed uterine leiomyomas presented for hysterectomy evaluation with her gynecologist. The patient had a Foley catheter placed due to urinary retention and hematuria, for which she underwent cystoscopic examination at an outside facility. A bladder mass was identified and biopsied, revealing a paraganglioma. She was thus referred to our institution for further evaluation of the bladder mass.

**Figure 1:**
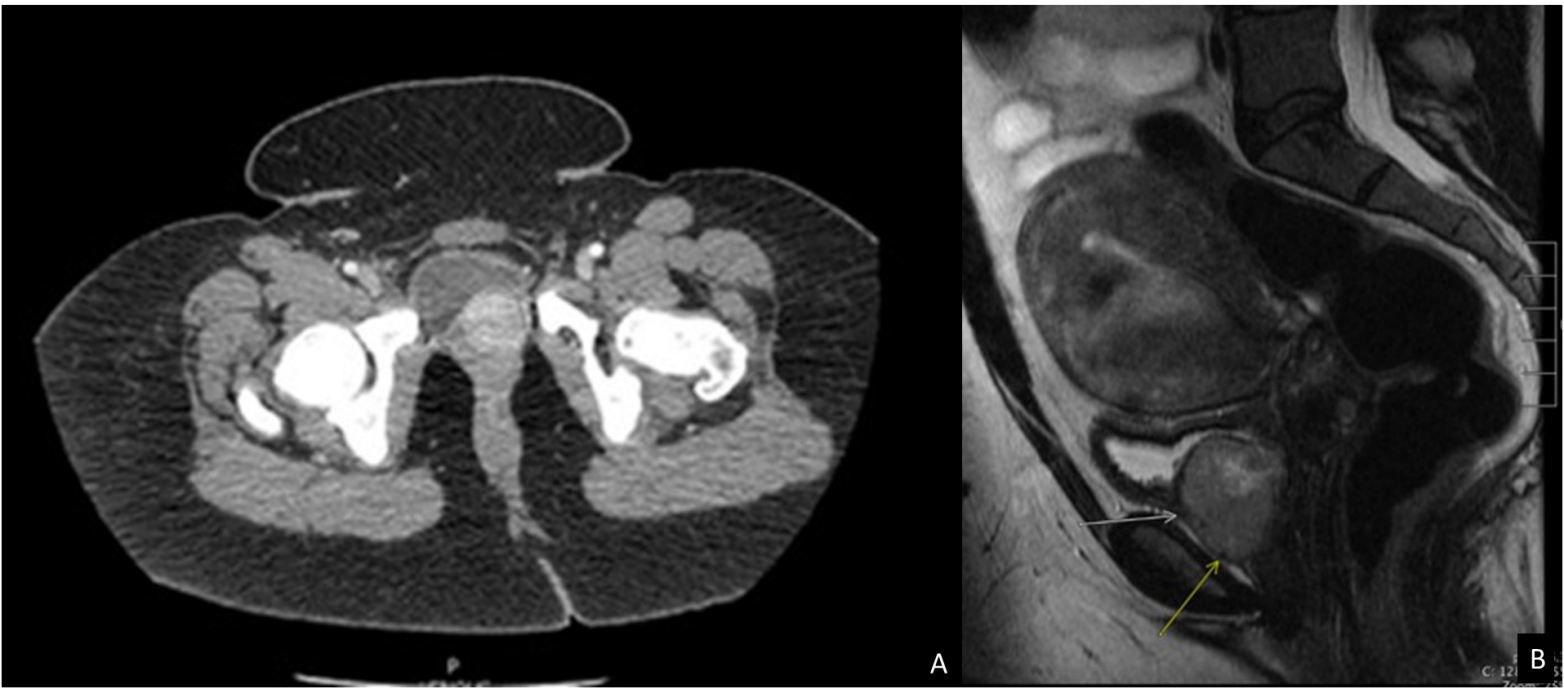
Imaging studies of the patient. **(A)** CT Intravenous pyelogram demonstrating an enhancing 3.7 cm bladder mass centered near the left ureterovesical junction without hydronephrosis or lymphadenopathy. **(B)** MRI pelvis and abdomen showing a well-defined 3.8 cm mass (arrows) in the anterior inferior aspect of the bladder abutting the urethra and vagina with a mild hypertense signal on T2 weighted image.

A CT intravenous pyelogram revealed an enhancing 3.7 cm bladder mass centered near the left ureterovesical junction without hydronephrosis (**Figure 1A**), as well as a hyperdense cardiac mass on the atrial septum, incompletely evaluated. A subsequent pelvic and abdominal MRI highlighted a well-defined heterogeneous soft tissue mass in the anterior inferior aspect of the bladder abutting the urethra and vagina, measuring 3.8x3.2x3.0 cm, that demonstrated a mild hyperintense signal on T2-weighted images (**Figure 1B**) as well as an isointense signal on T1-weighted images. This also showed a dominant 7.4 cm intramural leiomyoma in the anterior myometrial wall with a physiologic left ovarian cyst and no other cystic solid adnexal lesions. A transurethral resection of the bladder mass (TURBT) was scheduled.

In the meanwhile, based on the initial pathology results, the patient was also referred to endocrinology. Upon further interview at the endocrinology clinic, the patient reported excessive bleeding, both during her period and between her periods (menometrorrhagia), as well as episodes of spotting between menses. Bleeding episodes varied in quantity and quality; it is possible that the hematuria could have been masked by the abundant menometrorrhagia. She also reported fatigue and shortness of breath, which was attributed to severe iron deficiency secondary to abnormal uterine bleeding. Moreover, she complained of mild abdominal pain but denied episodic headaches, sweating, tremors, palpitations, anxiety, fevers, dysuria, or recreational drug use. The patient had no other past medical history or concomitant medication except for multivitamin supplements. Her family history was significant for stroke in her grandparents and father. There was no known family history of endocrine tumors.

Initial blood pressure and heart rate were 110/80 mmHg and 70–80 beats per minute respectively. However, ambulatory blood pressure monitoring revealed occasional values of 160/90 mmHg. At night, her blood pressures did not decrease. Of note, blood pressures were not monitored post-micturition. The physical exam revealed normal heart rate and rhythm with no murmur, rub, or gallop.

Further workup showed a slightly elevated plasma metanephrine level of 74 pg/mL (normal <57 pg/mL) with normal plasma normetanephrine and serum chromogranin levels. In her 24-hour urine studies, the levels of dopamine (564 mcg/24h; normal 52–480 mcg/24h) and metanephrine (305 mcg/24h; normal 58- 203 mcg/24h) were slightly elevated, while the normetanephrine level was normal.

There were no signs of pheochromocytoma or abdominal paragangliomas on abdominal MRI. To further evaluate synchronous paraganglioma, 68Ga-DOTATATE-PET/CT scan was performed before surgery and showed no evidence of metastatic disease, and no uptake of the hyperdense cardiac mass. Unfortunately, bladder visualization was limited due to the excreted tracer. Of note, both abdominal MRI and PET scans revealed suspicious breast nodules, however, biopsy showed fibroadenomatoid changes consistent with sclerosing adenosis with no evidence of malignancy.

To further characterize the cardiac mass, an echocardiogram was performed, which did not show the mass seen earlier on the CT scan. Further workup with cardiac MRI showed a well-circumscribed mass in the interatrial septum measuring 2.0 cm x 1.7 cm, with heterogeneous pattern in the late gadolinium enhancement sequences. A cardiologist was consulted for the left atrial mass, who concluded that it was most likely benign atrial myxoma based on MRI findings. No further treatment, including anticoagulation, was recommended.

To prevent an intraoperative hypertensive crisis, given occasional hypertension on ambulatory blood pressure monitoring, the patient received alpha blockade with doxazosin 2mg daily, which was up titrated to target blood pressure <130/80 mmHg supine and systolic blood pressure 90–110 mmHg upright, and to eliminate all occasionally high values. Her final dose before surgery was 8 mg twice daily. She then underwent TURBT for UBPGL resection. Doxazosin was continued post-operatively due to an elevated blood pressure reading. However, she decided to go home immediately after the surgery rather than stay overnight for monitoring. Therefore, she was discharged with doxazosin. Ten days later, she was seen by her cardiologist for a follow-up regarding her cardiac mass, and her dose was reduced. The patient had multiple rescheduled and missed appointments with endocrinologists. When she eventually followed up with endocrinology at three-month post-procedure, her dose was completely discontinued.

**Figure 2:**
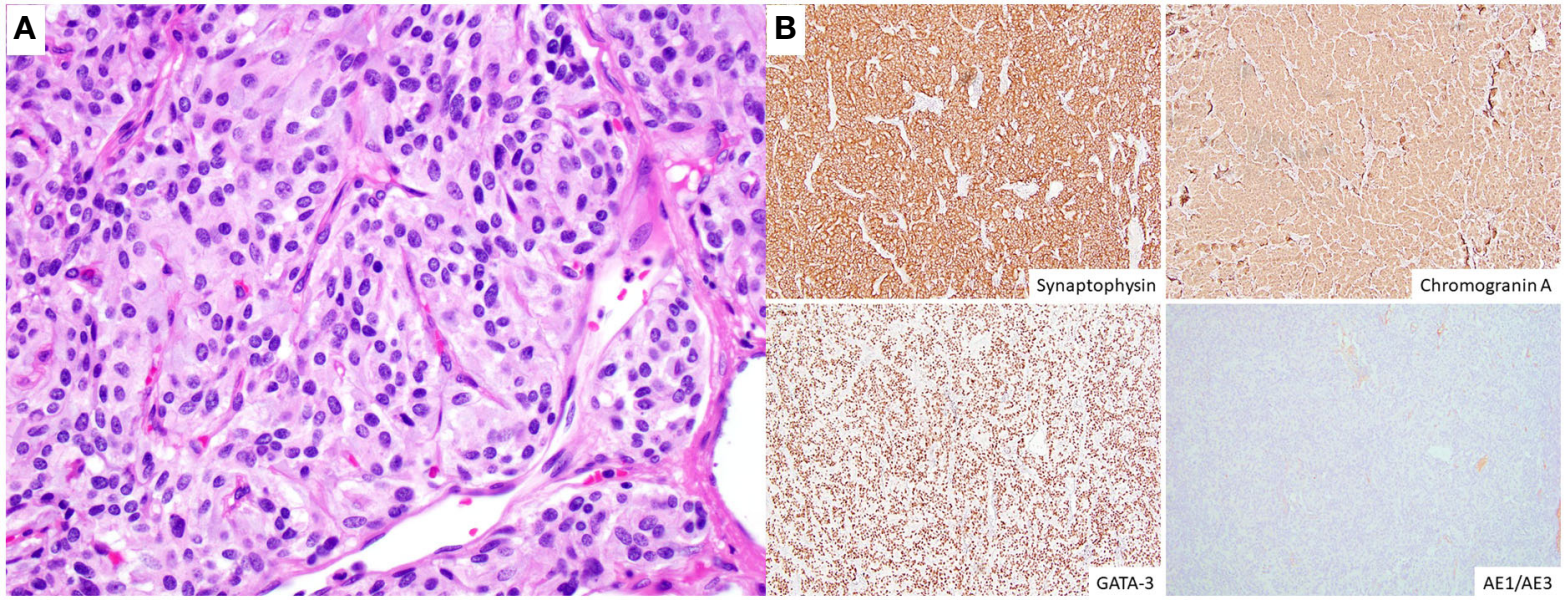
Histologic and immunohistochemical findings. **(A)** High magnification image (H&E, original magnification 400x) illustrating the tumor cells arranged in irregular nests surrounded by a fine capillary network, and showing moderate to abundant, eosinophilic cytoplasm, round to oval nuclei with finely granular (“salt and pepper”) chromatin and inconspicuous to absent nucleoli. **(B)** The tumor cells (immunohistochemistry, original magnification 100x) show diffuse cytoplasmic expression of synaptophysin and chromogranin A and nuclear immunoreactivity for GATA-3, with negative keratin (AE1/AE3) stains.

**Figure 3:**
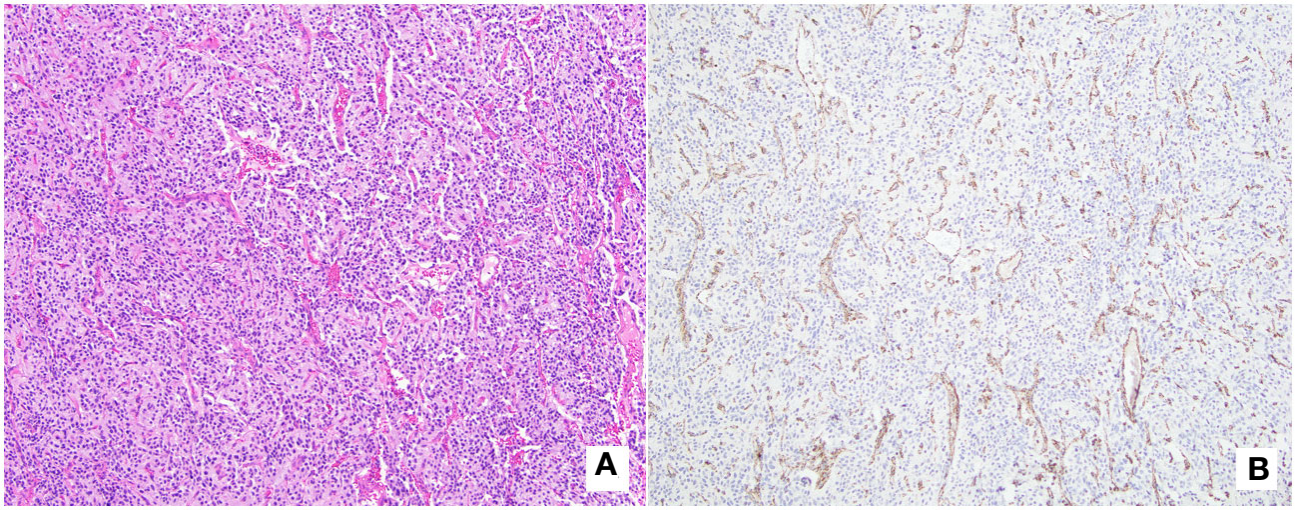
SDHB-immunochemistry with matching histology. **(A)** (H&E, original magnification 100x): Tumor is composed of cells with abundant eosinophilic cytoplasm arranged in a variable sized nested pattern, with associated thin capillary network. **(B)** (*SDHB* immunostain, original magnification 100x): Immunohistochemistry testing shows loss of *SDHB* protein expression in tumor cells, with appropriately staining internal control (endothelial cells outlining the vascular spaces, and stromal cells).

On pancystoscopy, the bladder mass appeared as a large (greater than 5 cm) endophytic mass of the left lateral and anterior walls that extended to the bladder neck. The gross specimen consisted of multiple irregularly and rectangularly shaped fragments of soft pinkish tissue that in total measured 7.0x6.0x1.0 cm in aggregate. Histologic sections showed again fragments of paraganglioma, composed of variably sized nests of eosinophilic cells with round to oval nuclei mostly arranged in a classic Zellballen pattern of growth, separated by a delicate fibrovascular network. No comedonecrosis or vascular invasion was identified. Immunohistochemical stains showed strong diffuse immunoreactivity with synaptophysin, chromogranin, and GATA-3, with negative keratin (AE1/AE3) and p63 stains (**Figure 2**). The ki-67 labeling index was greater than 3% (7.5% on average, as calculated over 1000 cells from hot spots in a representative section of the tumor). SDHB immunohistochemistry testing showed loss of stain in the tumor cells, with appropriately staining internal controls, represented by the endothelial and stromal cells (**Figure 3**). Overall, a diagnosis of primary UBPGL was made based on clinicopathologic findings.

**Figure 4:**
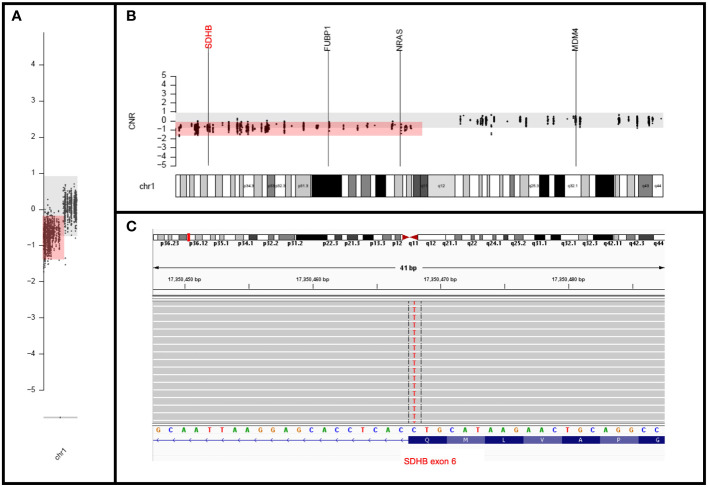
Molecular findings. **(A, B)** Copy number plots showing chromosome **(A)** and band level **(B)** loss of entire short arm of chromosome 1 with deletion of *SDHB*. **(C)** IGV view of the mutation in the last nucleotide of the *SDHB* exon 6 (negatively oriented gene) at the splice site.

Both germline and tumor genetic testing were undertaken as part of the clinical work-up. The germline testing was performed via the Ambry Genetics Custom-Next Cancer Panel (consisting of 85 genes) with RNA Insight and revealed a variant of unknown significance (VUS) in *MET* (c.816G>C, pQ272H). The tumor sample was sequenced by using our institution’s clinically validated 700-gene next-generation sequencing (NGS) panel known as UF Health Pathlabs Gatorseq700 NGS Screening Panel to evaluate for mutations and copy number variants. Briefly, genomic DNA extracted from the tumor was amplified using the GatorSeq700 NGS Panel and sequenced on the Novaseq 6000 to high uniform depth (targeting 500x coverage by non-PCR duplicate read pairs with >99% of exons at coverage >100x). Sequence data were processed using the genomic analysis application DRAGEN (enrichment version 3.9.5) with UCSC hg19-altaware as the reference genome. The mutation nomenclature was based on the convention recommended by the Human Genome Variation Society and interpretation was performed per clinical guidelines (9, 10). Tumor sequencing revealed a seemingly synonymous *SDHB* mutation (c.642G>A; p.Q214Q) with a deletion in the entire short arm of chromosome 1 spanning *SDHB* (**Figure 4**) and no evidence of loss of heterozygosity for the *MET* VUS. In silico analysis by multiple computational prediction tools supported the deleterious effect of the novel *SDHB* variant (c.642G>A; p.Q214Q), favoring a donor loss (see **Supplementary Data**).

Subsequently, the patient underwent a repeated bladder cystoscopy and an additional TURBT, which was negative for any residual tumor. Unfortunately, the patient has been lost to follow-up. We have contacted her in order to stress the importance of follow-up and to inquire about her blood pressure, menometrorrhagia, and repeated biochemical profiling.

## Discussion

Herein, we present a unique UBPGL case that illustrates the importance of a multidisciplinary team approach to navigate the clinical and imaging findings of this rare entity that was diagnosed during the workup of menometrorrhagia. In addition, we report a novel loss of function variant in *SDHB* which may be challenging to interpret given that there is no change in the predicted amino acid transcription if one solely focuses on the nucleotide changes in the codon (c.642G>A p.Q214Q).

PCCs are rare neuroendocrine tumors deriving from chromaffin cells of the adrenal glands, and approximately 10% of these neoplasms are in the extra-adrenal sites and are referred to as PGLs. Among them, UBPGLs are one of the rarest forms with only a couple of hundred cases reported in the literature (3, 5). They can manifest with a range of symptoms, although they may remain clinically silent. Between 30–53% of UBPGLs present with symptoms of catecholamine excess triggered by micturition, 35–47% present with hematuria, while about 3–10% are discovered on imaging incidentally (4–6). Up to 45% of the UBPGLs may be non-functioning (4–6). When there is clinical suspicion for a UBPGL, evaluation includes a cystoscopy and a CT scan of the abdomen and pelvis (11). In a retrospective study by Zhang et al. (12) looking at imaging characteristics of 16 UBPGL cases (9 of which were female patients), 13 patients underwent CT scans, which all exhibited slight hypoattenuation and moderate to marked enhancement of the bladder mass. There was only one case with leiomyomas on imaging, but it was unclear if that patient was symptomatic (12). No other reported cases of concurrent leiomyomas were noted in the literature. A multicentric study that investigated 110 patients diagnosed with UBPGL showed that only 37% were diagnosed prior to biopsy based on more characteristic symptoms (7). Overall, these studies highlight that PGLs may not be considered in the differential diagnosis of bladder masses during the initial workup.

Likewise, in our patient’s case, the history of leiomyomas and lack of significant symptoms of catecholamine excess made it challenging to initially consider a UBPGL in the differential diagnosis. Moreover, the slight increase in metanephrine level did not reach the level of significance (11) and could have been related to pain at the time. Considering the typical biochemical profile of paragangliomas against our findings, we concluded that our patient likely had a nonfunctioning bladder paraganglioma. Our case demonstrates that common tumors with expected symptoms, such as leiomyomas that present with abnormal uterine bleeding, may obscure findings of rare entities. The case also serves to encourage the clinicians to obtain detailed clinical histories and perform adequate work-up of incidental findings with a broad differential diagnosis.

Patients with UBPGLs present at a median age of 50 years and with a median tumor size of 2 cm (7). Hereditary PGLs manifest at an earlier age (approximately 15 years younger than average) and often present as multiple tumors (2, 7). Hereditary PGLs most commonly show germline mutations in *SDHB* and *VHL* (13, 14) while other genes such as *FH* (15) or *MET* (16) may rarely be involved. Studies have shown a strong correlation between loss of *SDHB* immunohistochemistry expression and *SDHx* mutations, with sensitivity and specificity both greater than 80% (15). Therefore, routine SDHB immunohistochemistry testing with PCCs or PGL tumors is a highly effective and rather inexpensive surrogate marker for *SDHx* mutations, thus representing a valuable screening tool for determining the necessity of germline testing in these tumors. The World Health Organization (WHO) classification of endocrine tumors considers *SDHB* immunohistochemistry “essential” in the histopathologic diagnosis of parasympathetic (most head and neck) PGLs and “desirable” in sympathetic PGLs (17). Our patient was relatively young and had multiple organ masses based on imaging. In addition, her UBPGL’s pathology had a loss of *SDHB* expression. Although these findings may initially raise the potential for germline syndrome, she had only one histologically confirmed PGL from her urinary bladder. Her breast lesions were biopsy proven to be non-malignant and her cardiac tumor was favored to be a myxoma based on MRI and PET findings. The immunohistochemical loss of SDHB was accompanied by the tumor NGS testing finding of a novel *SDHB* variant (c.642G>A; p.Q214Q), however, germline testing excluded this variant as being hereditary. Of note, there was a germline *MET* variant (c.816G>C, pQ272H) which was interpreted by the reference laboratory and clinical team as a VUS (also supported by ClinVar entries (18) Variant ID: 1401743).

Overall, despite the initial findings pointing towards a hereditary syndrome, a thorough workup by multidisciplinary teams, including additional germline and somatic (tumor) molecular testing, favored this tumor to be a sporadic UBPGL with a novel somatic *SDHB* variant. Even in cases of *SDH*-deficient neoplasia where no germline mutation was found, surveillance and further follow-up for other *SDH*-deficient neoplasms are still recommended (19). Even though, a germline pathogenic variant was not found in our patient, future follow-up with 68Ga-DOTATATE-PET/CT will be pursued, to continue to monitor for metastases and other neoplasms, based on other risk factors present in this case, including the young (41 years) age at presentation, the relatively large UBPGL size (3.8 cm on imaging), and the tumorigenic mutation in *SDHB.*

The *SDHB* (c.642G>A; p.Q214Q) variant itself raised a molecular diagnostic challenge in this case due to its seemingly synonymous change in the protein nomenclature. However, it occurred at the last nucleotide of exon 6 (**Figure 4**), which was predicted to result in a splice site effect (20) in this gene with loss of function variants considered pathogenic. This amino acid position is highly conserved across species and this variant has not been observed in population databases. Different mutations at the same nucleotide position have been reported as likely pathogenic; these include c.642G>T (2 ClinVar entries, Variation ID: 480788) and c.642G>C (reported in a patient with a malignant paraganglioma) (21) and listed in Human Gene Mutation Database (CM065460). In addition, the deleterious effect of this variant was further supported by in silico analysis by multiple prediction tools (see **Supplementary Material**). Lastly, the WHO for Genetic Tumour Syndromes recommends performing *SDHB* immunohistochemistry for evaluation of a VUS in *SDHB*, with a loss implying pathogenicity (19).

In combination with the patient’s tumor histology and the loss of immunohistochemical expression of *SDHB*, this somatic variant is ultimately interpreted as a likely pathogenic somatic variant (confirmed by germline testing) and the potential driver of this presumed sporadic tumor. Of note, there was a loss of heterozygosity of *SDHB* by deletion of the entire short arm of chromosome 1, which is a common mechanism for biallelic inactivation in the setting of somatic mutations, first described in this tumor type by van Nederveen et al. (22).

Surgical intervention for UBPGLs is typically personalized to the patient due to the lack of prospective research and clear criteria for malignancy prediction. It is known that there are no absolute histologic criteria or single biomarkers to reliably predict the biological behavior of PGLs or PCCs, and multi-parameter scoring systems have been proposed. The GAPP (Grading system for Adrenal Pheochromocytoma and Paraganglioma) (23) and modified GAPP (24) have shown predictive value to varying degrees, and they may be particularly useful to “rule out” potentially aggressive behavior rather than “rule it in” for risk stratification. The current patient’s tumor characteristics would add up to a GAPP score of 4 (“intermediate risk”). However, the risk stratification also depends on extra-adrenal location, patient age, number of tumors, and evidence of metastasis. A comprehensive approach should include clinical, biochemical, molecular, and pathological assessments (25) Short-term safety for procedures like transurethral resection and cystectomies is documented, yet long-term outcome data remains limited. Usually, UBPGLs are initially treated with either cystectomy or TURBT (7). Repeated surgery is sometimes required, especially in those of younger age (<5 years old) and large tumor size (>1 cm). Patients with incomplete resection or higher tumor stages (‗ T3) are at higher risk of recurrence, metastases, and death when compared to those with lower stages (26). Although the prognosis is usually good, about 8% of patients present with synchronous metastases, and 22% of patients develop metachronous metastases. These patients tend to be young, have a large UBPGL size, and have a high degree of catecholamine excess (4). Our patient was asymptomatic with negative repeated bladder resection at 3 months; however, our study is limited due to a lack of long-term follow-up.

This unique case of a UBPGL showcases multiple layers of diagnostic challenges starting with the patient’s initial abnormal uterine bleeding and leiomyoma masking the symptoms of this rare entity. The patient’s relatively young age, additional findings throughout the clinical workup of other masses at different sites, and the loss of *SDHB* immunohistochemistry in the UBPGL raised the potential for a germline syndrome, which was then excluded with germline and tumor genetic testing. Lastly, the somatic *SDHB* variant highlights the importance of positional effects at splice sites in “seemingly synonymous” variants in molecular diagnostics. Overall, a comprehensive approach combining multiple layers of data, from clinical history to molecular findings with multidisciplinary teamwork, is essential for diagnosing rare and challenging cases like ours.

## Data availability statement

The original contributions presented in the study are included in the article/**Supplementary Material**. Further inquiries can be directed to the corresponding authors.

## Ethics statement

Written informed consent was obtained from the individual(s) for the publication of any potentially identifiable images or data included in this article.

## Author contributions

TN: Writing – original draft, Writing – review & editing. ZO: Writing – review & editing, Investigation, Supervision, Formal Analysis, Conceptualization. SS: Writing – review & editing. UP: Investigation, Writing – review & editing. PC: Investigation, Writing – review & editing. LB: Investigation, Writing – review & editing. SF: Investigation, Writing – review & editing, Resources. HG: Conceptualization, Investigation, Supervision, Validation, Writing – review & editing, Project administration, Resources. JP: Investigation, Supervision, Writing – review & editing.

## References

[1] Erickson D Kudva YC Ebersold MJ Thompson GB Grant CS van Heerden JA. Benign paragangliomas: clinical presentation and treatment outcomes in 236 patients. J Clin Endocrinol Metab. (2001) 86:5210–6. doi: 10.1210/jcem.86.11.8034 11701678

[2] Neumann HPH Young WF Eng C. Pheochromocytoma and paraganglioma. N Engl J Med. (2019) 381:552–65. doi: 10.1056/NEJMra1806651 31390501

[3] Purnell S Sidana A Maruf M Grant C Agarwal PK. Genitourinary paraganglioma: demographic, pathologic, and clinical characteristics in the surveillance, epidemiology, and end results (SEER) database (2000–2012). Urol Oncol. (2017) 35:457.e9–457.e14. doi: 10.1016/j.urolonc.2017.02.006 PMC5476479 28325651

[4] Beilan JA Lawton A Hajdenberg J Rosser CJ. Pheochromocytoma of the urinary bladder: a systematic review of the contemporary literature. BMC Urol. (2013) 13:22. doi: 10.1186/1471-2490-13-22 23627260 PMC3654956

[5] Li M Xu X Bechmann N Pamporaki C Jiang J Propping S. Differences in clinical presentation and management between pre- and postsurgical diagnoses of urinary bladder paraganglioma: is there clinical relevance? A systematic review. World J Urol. (2022) 40:385–90. doi: 10.1007/s00345-021-03851-x PMC8921018 34655306

[6] Park S Kang SY Kwon GY Kwon JE Kim SK Kim JY. Clinicopathologic characteristics and mutational status of succinate dehydrogenase genes in paraganglioma of the urinary bladder: A multi-institutional Korean study. Arch Pathol Lab Med. (2016) 141:671–7. doi: 10.5858/arpa.2016-0403-OA 27819762

[7] Yu K Ebbehøj AL Obeid H Vaidya A Else T Wachtel H. Presentation, management, and outcomes of urinary bladder paraganglioma: results from a multicenter study. J Clin Endocrinol Metab. (2022) 107:2811–21. doi: 10.1210/clinem/dgac427 PMC9516048 35882219

[8] Su T Yang Y Jiang L Xie J Zhong X Wu L. SDHB immunohistochemistry for prognosis of pheochromocytoma and paraganglioma: A retrospective and prospective analysis. Front Endocrinol (Lausanne). (2023) 14:1121397. doi: 10.3389/fendo.2023.1121397 37008946 PMC10061060

[9] Li MM Datto M Duncavage EJ Kulkarni S Lindeman NI Roy S. Standards and guidelines for the interpretation and reporting of sequence variants in cancer. J Mol Diagn. (2017) 19:4–23. doi: 10.1016/j.jmoldx.2016.10.002 27993330 PMC5707196

[10] Richards S Aziz N Bale S Bick D Das S Gastier-Foster J. Standards and guidelines for the interpretation of sequence variants: a joint consensus recommendation of the American College of Medical Genetics and Genomics and the Association for Molecular Pathology. Genet Med. (2015) 17:405–24. doi: 10.1038/gim.2015.30 PMC4544753 25741868

[11] Lenders JWM Duh QY Eisenhofer G Gimenez-Roqueplo AP Grebe SKG Murad MH. Pheochromocytoma and paraganglioma: an endocrine society clinical practice guideline. J Clin Endocrinol Metab. (2014) 99:1915–42. doi: 10.1210/jc.2014-1498 24893135

[12] Zhang J Bai X Yuan J Zhang X Xu W Ye H. Bladder paraganglioma: CT and MR imaging characteristics in 16 patients. Radiol Oncol. (2021) 56:46–53. doi: 10.2478/raon-2021-0055 34973050 PMC8884856

[13] de Tersant M Généré L Freyçon C Villebasse S Abbas R Barlier A. Pheochromocytoma and paraganglioma in children and adolescents: experience of the French society of pediatric oncology (SFCE). J Endocr Soc. (2020) 4:bvaa039. doi: 10.1210/jendso/bvaa039 32432211 PMC7217277

[14] Virgone C Andreetta M Avanzini S Chiaravalli S De Pasquale D Crocoli A. Pheochromocytomas and paragangliomas in children: Data from the Italian Cooperative Study (TREP). Pediatr Blood Cancer. (2020) 67:e28332. doi: 10.1002/pbc.28332 32491270

[15] Udager AM Magers MJ Goerke DM Vinco ML Siddiqui J Cao X. The utility of SDHB and FH immunohistochemistry in patients evaluated for hereditary paraganglioma-pheochromocytoma syndromes. Hum Pathol. (2018) 71:47–54. doi: 10.1016/j.humpath.2017.10.013 29079178

[16] Toledo RA Qin Y Cheng ZM Gao Q Iwata S Silva GM. Recurrent mutations of chromatin remodeling genes and kinase receptors in pheochromocytomas and paragangliomas. Clin Cancer Res. (2016) 22:2301–10. doi: 10.1158/1078-0432.CCR-15-1841 PMC4854762 26700204

[17] WHO Classification of Tumours Editorial Board. Who Classification of Tumours: Endocrine and Neuroendocrine Tumours. 5 ed. Lyon (France: International Agency for Research on Cancer (2022).

[18] VCV001401743.8 - ClinVar - NCBI. Available at: https://www.ncbi.nlm.nih.gov/clinvar/variation/1401743/?oq=1401743&m=NM_000245.4(MET):c.816G%3EC%20(p.Gln272His).

[19] WHO Classification of Tumours Editorial Board. Who Classification of Tumours: Genetic Tumour Syndromes Website beta version. International Agency for Research on Cancer (2024). Available at: https://publications.iarc.fr. cited 2024 03/31/2024.

[20] Cartegni L Chew SL Krainer AR. Listening to silence and understanding nonsense: exonic mutations that affect splicing. Nat Rev Genet. (2002) 3:285–98. doi: 10.1038/nrg775 11967553

[21] Jochmanova I Wolf KI King KS Nambuba J Wesley R Martucci V. SDHB-related pheochromocytoma and paraganglioma penetrance and genotype-phenotype correlations. J Cancer Res Clin Oncol. (2017) 143:1421–35. doi: 10.1007/s00432-017-2397-3 PMC5505780 28374168

[22] van Nederveen FH Korpershoek E Lenders JWM de Krijger RR Dinjens WNM. Somatic SDHB mutation in an extraadrenal pheochromocytoma. N Engl J Med. (2007) 357:306–8. doi: 10.1056/NEJMc070010 17634472

[23] Kimura N Takayanagi R Takizawa N Itagaki E Katabami T Kakoi N. Pathological grading for predicting metastasis in phaeochromocytoma and paraganglioma. Endocr Relat Cancer. (2014) 21:405–14. doi: 10.1530/ERC-13-0494 24521857

[24] Koh JM Ahn SH Kim H Kim BJ Sung TY Kim YH. Validation of pathological grading systems for predicting metastatic potential in pheochromocytoma and paraganglioma. PLoS One. (2017) 12:e0187398. doi: 10.1371/journal.pone.0187398 29117221 PMC5678867

[25] Kimura N Takekoshi K Naruse M. Risk stratification on pheochromocytoma and paraganglioma from laboratory and clinical medicine. J Clin Med. (2018) 7:242. doi: 10.3390/jcm7090242 30150569 PMC6162838

[26] Cheng L Leibovich BC Cheville JC Ramnani DM Sebo TJ Neumann RM. Paraganglioma of the urinary bladder. Cancer. (2000) 88:844–52. doi: 10.1002/(SICI)1097-0142(20000215)88:4<844::AID-CNCR15>3.0.CO;2-I 10679654

