# Supplementary material for: Case report: A novel somatic SDHB variant in a patient with bladder paraganglioma
Source: Front Endocrinol (Lausanne). 2024 Jun 7;15:1386285. doi: 10.3389/fendo.2024.1386285 (PMC11190172; doi:10.3389/fendo.2024.1386285)
Supplement: Supplementary file 1 [file DataSheet_1.pdf]

The clinical sequence is the following:

1. The patient initially complained of menometrorrhagia which was presumed to be secondary to uterine leiomyomata. She presented initially for possible hysterectomy with her gynecologist.
2. A Foley catheter was placed due to the patient's urinary retention and hematuria. She then underwent a cystoscopy and TURBT for biopsy. Imaging with CT pyelogram and MRI Abdomen/Pelvis revealed leiomyomata and a bladder mass.
3. Pathology assessment of the biopsy determined the bladder mass to be a paraganglioma
4. The patient was referred to endocrinologist and was then started on an alpha-1 blocking agent. Abdominal MRI and 68Ga-DOTATATE-PET/CT were also recommended.
5. The patient underwent a successful resection of the bladder mass with a second TURBT procedure. Pathologic assessment of specimens obtained intra-operatively determined these to be fragments of a bladder paraganglioma
6. Tumor sequencing revealed a seemingly synonymous SDHB mutation (c.642G>A; p.Q214Q) with a deletion in the entire short arm of chromosome 1 spanning SDHB and no evidence of loss of heterozygosity for the MET VUS.

In silico analysis by multiple computational prediction tools supports the deleterious effect of the novel SDHB variant (c.642G>A; p.Q214Q), favoring a donor loss

- MaxEntScan: Likely disrupted (splicing efficiency predicted to be decreased by a MaxEntScan score decrease of 41% (from 10.07 to 5.85))(1)
- Combined Annotation Dependent Depletion (CADD) score: 25.3 (Deleterious) (2)
- Mutation taster: disease-causing (“alteration within used splice site, likely to disturb normal splicing”) (3)
- Splice AI Donor Loss score: 0.76 (higher end of a “recommended”/moderate score) (4)

References

1. Yeo G, Burge CB. Maximum entropy modeling of short sequence motifs with applications to RNA splicing signals. J Comput Biol. 2004;11(2–3):377–94.

2. Rentzsch P, Schubach M, Shendure J, Kircher M. CADD-Splice-improving genome-wide variant effect prediction using deep learning-derived splice scores. Genome Med. 2021 Feb 22;13(1):31.

3. Steinhaus R, Proft S, Schuelke M, Cooper DN, Schwarz JM, Seelow D. MutationTaster2021. Nucleic Acids Res. 2021 Jul 2;49(W1):W446–51.

4. de Sainte Agathe JM, Filser M, Isidor B, Besnard T, Gueguen P, Perrin A, et al. SpliceAI-visual: a free online tool to improve SpliceAI splicing variant interpretation. Human Genomics [Internet]. 2023 Feb 10 [cited 2024 Apr 24];17(1):7. Available from: https://doi.org/10.1186/s40246-023-00451-
